# Overcoming Anatomical Challenges: Epidural Blood Patch and Plica Mediana Dorsalis Case Report

**DOI:** 10.7759/cureus.76501

**Published:** 2024-12-28

**Authors:** Isaiah Florence, Steven Yap, Ram Manchandani, Artem Babayan

**Affiliations:** 1 Anesthesiology, South Brooklyn Health, Brooklyn, USA; 2 Anesthesiology, South Brooklyn Health, New York, USA

**Keywords:** case report, epidural blood patch, plica mediana dorsalis, post-dural puncture headache, spinal csf leak

## Abstract

The efficacy of epidural blood patch (EBP) is highly variable, and often, clinicians are unable to identify the underlying reasons for treatment failure. A 36-year-old parturient underwent a "blind" epidural blood patch (EBP) without image guidance but failed to obtain relief from a postural headache related to the labor epidural. During the second EBP, an intact plica mediana dorsalis (PMD) was visualized in the anterior-posterior fluoroscopic view after injection of contrast, and autologous blood was injected on both sides of the PMD, leading to the complete resolution of headache symptoms. This case study presents a unique finding, potentially the first identification of an intact PMD as the etiology behind the failure of an EBP to provide symptomatic relief. The first EBP was performed without image guidance, which would have allowed the identification of an intact PMD. Fluoroscopic guidance, utilizing anterior-posterior and lateral views in combination with an adequate volume of contrast to confirm bilateral spread, enables the identification of anatomic variants such as an intact PMD.

## Introduction

Post-dural puncture headache (PDPH) is the most common serious complication resulting from lumbar punctures and neuraxial anesthesia. Its overall incidence is approximately 0.3% to 3% with typical obstetric anesthesiology practices [1-3]. Epidural blood patches are commonly indicated for the treatment of PDPH, particularly when conservative management (e.g., hydration, analgesics, and caffeine) fails, and the headache significantly impairs the patient's daily functioning. The procedure involves the injection of 7mL to 25mL of autologous blood into the epidural space to seal the dural hole and alleviate the headache [4].

Epidural blood patches (EBP) are often performed "blind" without the aid of imaging guidance. This procedure is conducted based on anatomic landmarks and clinician experience and typically has a success rate of around 69% for providing durable relief from severe headache symptoms [4]. 

Targeted or guided epidural blood patches use imaging techniques such as fluoroscopy or ultrasound to confirm the appropriate spread of contrast or blood injected into the epidural space over the area of cerebrospinal fluid (CSF) leak, typically corresponding to the spinal level where the neuraxial procedure was performed and offer a success rate as high as 87% [5,6]. Despite the increased precision of targeted EBP, there is limited discussion of the etiologies behind why targeted EBPs still fail, including the presence of anatomical landmarks.

## Case presentation

A 36-year-old female, G4, P2, AB 2, experienced an uneventful normal vaginal delivery with epidural analgesia administered at the L2-L3 level, utilizing a 19-gauge catheter inserted through a 17-gauge Touhy needle. Several hours after discontinuation of epidural analgesia, she developed a severe positional headache consistent with PDPH. Neurological examination was otherwise grossly normal, with no visual changes, photophobia, tinnitus, or nuchal rigidity. Her headache persisted despite two days of conservative management with non-steroidal anti-inflammatory drugs and three liters of lactated ringers over a 24-hour period. The patient's consent was obtained for publication.

The first EBP was performed using fluoroscopic guidance at the level of L3-L4, with skin puncture sites providing guidance as to the level of CSF leakage. In the lateral fluoroscopic view, 0.5mL of Iopamidol M180 was injected to confirm entry into the epidural space. The bilateral spread could not be confirmed, as the anterior-posterior fluoroscopic view was not performed. 20mL of autologous blood was then injected, and the patient's headache immediately resolved. She was then discharged home. 

**Figure 1:**
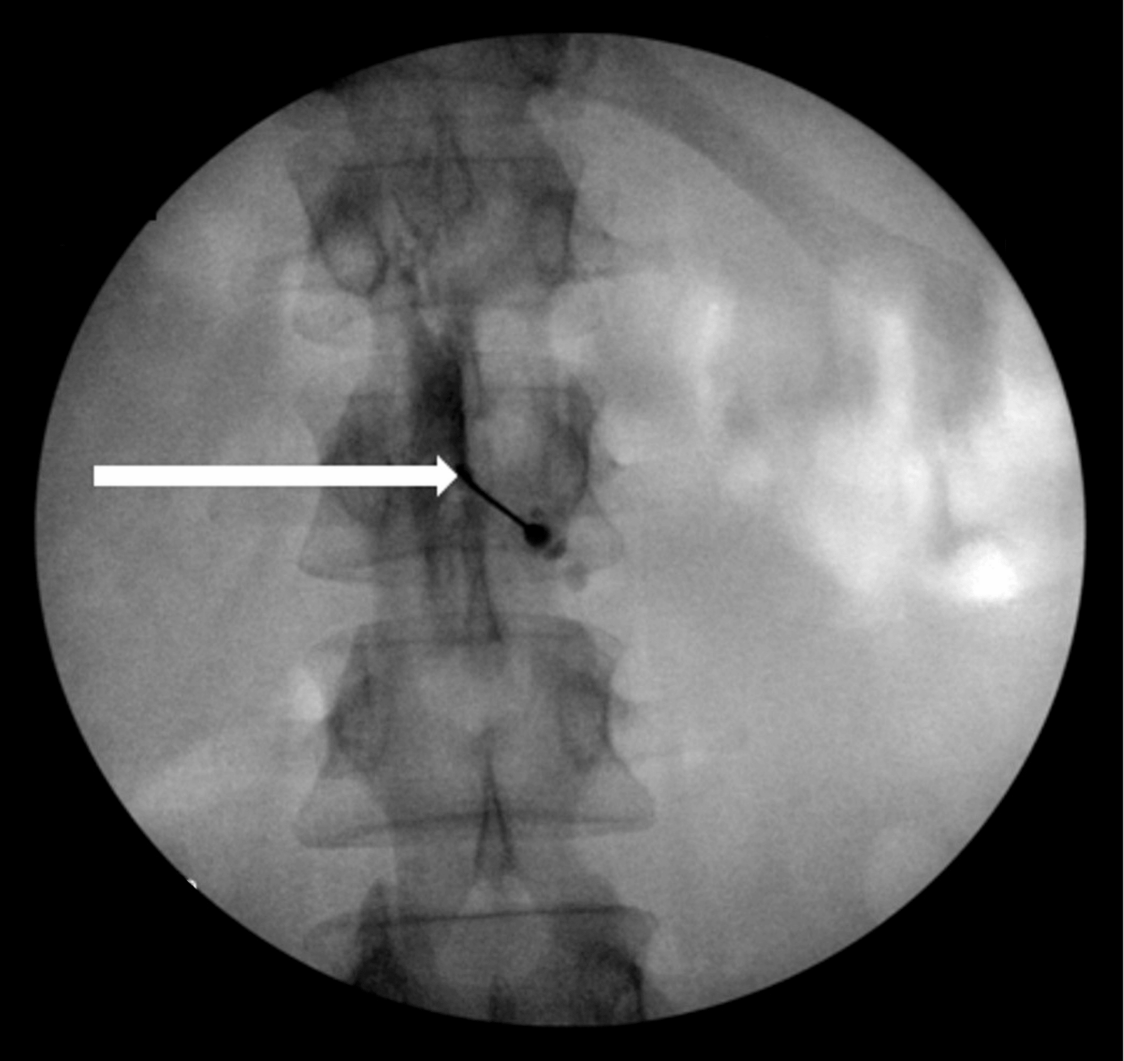
AP view showing unilateral spread of contrast with linear flow obstruction at the midline AP - anterior-to-posterior

The patient returned two days later, reporting recurrent headache symptoms identical to the initial presentation. A second epidural blood patch was administered with a 20-gauge Touhy needle at the L2-L3 level using fluoroscopic guidance. Upon injection of 1cc of Iopamidol M180 contrast, it was noted that the patient had a plica mediana dorsalis (PMD) as the contrast flowed only to the left of the midline (Figure 1). 

**Figure 2:**
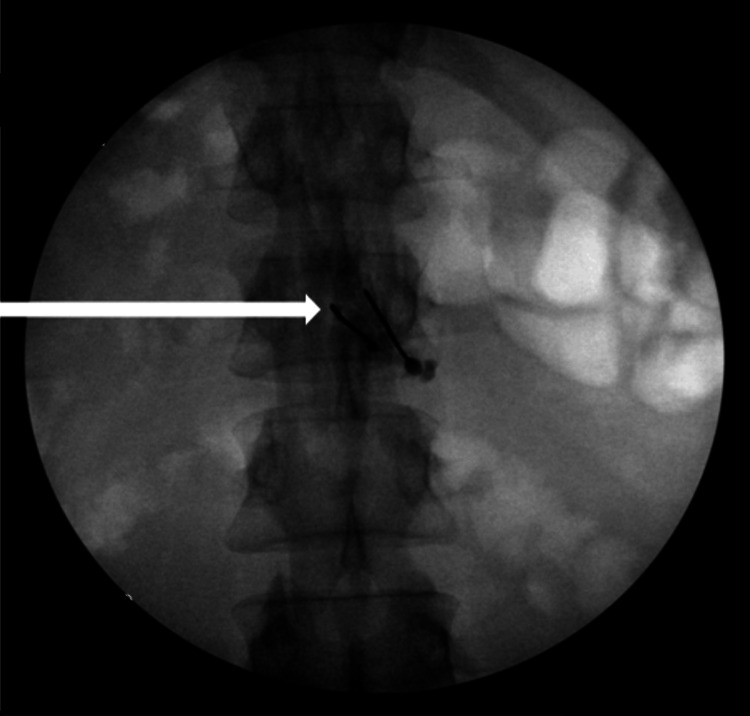
Placement of two Touhy needles demonstrating bilateral contrast spread in epidural space

A second 20-gauge Touhy needle was then placed at the L2 lumbar level on the right side of the midline, and a repeat injection of 1mL of Iopamidol M180 contrast filled the deficit on the right side of the epidural space (Figure 2).

10cc of autologous blood was injected through each needle. The patient was discharged without a headache, and she expressed satisfaction with no recurrence of headache symptoms or side effects during two years of follow-up.

## Discussion

The mechanism by which EBPs relieve PDPH symptoms is likely multifactorial [7-10]. In the initial hours after the procedure, the injected blood compresses the thecal sac, increasing intracranial pressure and causing reflex vasoconstriction. A sustained therapeutic response is believed to result from the formation of a blood clot at the site of the dural puncture, effectively sealing the tear or hole and allowing CSF pressure to normalize.

Blind EBPs are associated with relatively higher failure rates of 30% to 56% [5,11]. Targeted EBPs, guided with fluoroscopy and injection of contrast, have been shown to significantly improve efficacy, with success rates between 84.8 and 87% [5,6]. This improvement is attributed to epidural placement at the intended level with contrast spread in the epidural space. Additionally, fluoroscopic visualization of blood spread in the epidural space over the suspected dural puncture site, achieved by injecting a 4:1 mixture of autologous blood and contrast under fluoroscopic guidance, may further enhance the success rate [12]. Lastly, this case report highlights the importance of performing an anterior-posterior fluoroscopy view to confirm the bilateral spread of contrast.

This case highlights a rare finding wherein fluoroscopy and contrast injection were utilized to identify an intact PMD [13], which may have significantly contributed to the failure of the initial EBP to provide relief. It also emphasizes the importance of obtaining an anterior-posterior view to visualize the contrast spread within the epidural space, along with the necessity of injecting an adequate volume of contrast to further enhance the success rate of targeted EBPs. The use of fluoroscopic guidance facilitated the identification of a midline raphe that would have hindered the even distribution of the blood patch bilaterally in the epidural space. Consequently, it required the use of two different injection sites on both sides of the PMD to overcome this anatomical barrier. Limitations may include the possibility that the PMD may not have been the cause of failure of the first EBP, as the spread of blood can exhibit nonspecific variations between patients. However, this is unlikely, considering the unusually linear blockage of contrast spread.

## Conclusions

This case illustrates the value of fluoroscopy and contrast injection in identifying an intact PMD, which may have contributed to the failure of the initial epidural blood patch (EBP) to provide relief. Fluoroscopy helped reveal a midline raphe, likely caused by Hoffman’s ligament, that impeded the even distribution of the blood patch. To overcome this barrier, two different injection sites were used on either side of the raphe. While the role of the PMD in the failure of the first EBP is uncertain, the findings emphasize the importance of contrast spread and fluoroscopic guidance for improving EBP success rates.

## References

[1] Buddeberg BS, Bandschapp O, Girard T (2019). Post-dural puncture headache. Minerva Anestesiol.

[2] Hughes D, Simmons SW, Brown J, Cyna AM (2003). Combined spinal-epidural versus epidural analgesia in labour. Cochrane Database Syst Rev.

[3] Guglielminotti J, Landau R, Ing C, Li G (2021). Temporal trends in the incidence of post-dural puncture headache following labor neuraxial analgesia in the United States, 2006 to 2015. Int J Obstet Anesth.

[4] Banks S, Paech M, Gurrin L (2001). An audit of epidural blood patch after accidental dural puncture with a Tuohy needle in obstetric patients. Int J Obstet Anesth.

[5] Kranz PG, Malinzak MD, Amrhein TJ, Gray L (2017). Update on the diagnosis and treatment of spontaneous intracranial hypotension. Curr Pain Headache Rep.

[6] Özütemiz C, Köksel YK, Huang H, Rubin N, Rykken JB (2019). The efficacy of fluoroscopy-guided epidural blood patch in the treatment of spontaneous and iatrogenic cerebrospinal fluid leakage. Eur Radiol.

[7] Beards SC, Jackson A, Griffiths AG, Horsman EL (1993). Magnetic resonance imaging of extradural blood patches: appearances from 30 min to 18 h. Br J Anaesth.

[8] Duffy PJ, Crosby ET (1999). The epidural blood patch. Resolving the controversies. Can J Anaesth.

[9] Gaiser R, McDonald S, O'Flaherty J (2014). Epidural blood patch: a review of the literature. Reg Anesth Pain Med.

[10] Buvanendran A, Ball S, Tumin D (2007). Mechanism of action of the epidural blood patch in post-dural puncture headache. Anesth Analg.

[11] Sprigge JS, Harper SJ (2008). Accidental dural puncture and post dural puncture headache in obstetric anaesthesia: presentation and management: a 23-year survey in a district general hospital. Anaesthesia.

[12] Kawaguchi M, Hashizume K, Watanabe K, Inoue S, Furuya H (2011). Fluoroscopically guided epidural blood patch in patients with postdural puncture headache after spinal and epidural anesthesia. J Anesth.

[13] Savolaine ER, Pandya JB, Greenblatt SH, Conover SR (1988). Anatomy of the human lumbar epidural space: new insights using CT-epidurography. Anesthesiology.

